# Effect of vitamin D receptor gene TaqI polymorphism on vitamin D levels and biochemical, inflammatory, and oxidative stress markers in individuals with obesity

**DOI:** 10.1007/s11033-026-12022-3

**Published:** 2026-05-29

**Authors:** Laura Smolski dos Santos, Gênifer Erminda Schreiner, Carolina Pereira de Oliveira, Camila Berny Pereira, Silvia Muller de Moura Sarmento, Debora Alejandra Vasquez Rubio, Lyana Feijoo Berro Pivetta, Sandra Mary Lima Vasconcelos, Fernanda Barbisan, Vanessa Rosa Retamoso, Jacqueline da Costa Escobar Piccoli, Vanusa Manfredini

**Affiliations:** 1 Postgraduate Program in Biochemistry, Federal University of Pampa, Uruguaiana, Rio Grande do Sul Brazil; 2 Multicenter Graduate Program in Physiological Sciences, Federal University of Pampa, Uruguaiana, Rio Grande do Sul Brazil; 3 Postgraduate Program in Nutrition, Federal University of Alagoas, Maceió, Alagoas Brazil; 4 Postgraduate Program in Gerontology, Federal University of Santa Maria, Santa Maria, Rio Grande do Sul Brazil

**Keywords:** Obesity, Vitamin D, Inflammation, Oxidative stress, TaqI polymorphism

## Abstract

Obesity, characterized by excessive accumulation of body fat, is associated with reduced vitamin D levels. Several polymorphisms of the vitamin D receptor exist, such as TaqI (rs731236), although their association with obesity remains inconsistent. The aim of this study was to evaluate the effect of TaqI polymorphism on biomarkers in individuals with obesity. The study has been approved by the Research Ethics Committee of the Federal University of Pampa (UNIPAMPA), under approval number 5.308.525. A total of 275 individuals were included in the study, divided into groups according to Body Mass Index: eutrophic, overweight, and individuals with obesity. Blood samples and anthropometric data were collected for analysis and 246 individuals were genotyped for the VDR gene TaqI polymorphism. Results showed that vitamin D levels were higher in individuals with CC genotype compared with those with TT genotype. A decrease in biochemical parameter levels was observed in the obesity group. In contrast, inflammatory and oxidative stress markers were increased in the overweight and obesity groups. Additionally, TBARS, IL-8, IL-10, and CRP were identified as independent risk factors for obesity, where vitamin D was identified as an independent protective factor. Although TaqI polymorphism was not an independent risk factor for obesity, our results emphasize the relevance of inflammatory and oxidative stress biomarkers in obesity risk assessment. Moreover, the modulation of vitamin D levels by genotype reinforces its potential as a target for preventive and therapeutic interventions due to its protective role.

## Introduction

Obesity, classically defined as the excessive accumulation of body fat, is considered a public health problem due to the significant increase in cases worldwide, and is also a risk factor for chronic diseases such as type 2 diabetes mellitus, systemic arterial hypertension, and cardiovascular diseases [1–3]. Of multifactorial origin, obesity has both lifestyle-dependent and lifestyle-independent factors. Environmental factors include inadequate diet and lack of physical activity; however, genetic factors are also described, which can influence the increased storage and distribution of body fat [4]. There is an inverse association between body mass index (BMI) and serum vitamin D levels, as a BMI greater than 30 kg/m^2^ is associated with vitamin D deficiency, meaning that individuals with obesity require higher than normal intakes of vitamin D to reach levels comparable to those with normal weight [5]. This occurs due to the sequestration of vitamin D in adipose tissue, as it is a fat-soluble vitamin. After being absorbed, some of it is captured and stored in adipose tissue, leading to decrease in its bioavailability [6–7].

Vitamin D can be found in the form of ergocalciferol (vitamin D2) or cholecalciferol (vitamin D3), and plays an important role as nutrient, and its deficiency is associated with some chronic diseases, such as osteoporosis, cardiovascular disease, and obesity [8–9]. While vitamin D2 can be synthesized in plants, vitamin D3 is synthesized from the skin in humans and animals through ultraviolet beta (UVB) radiation. Obtainable from diet, vitamin D2 can be found in plants and fungi, and D3 in meat, fish, and eggs [10]. Both forms of vitamin D obtained from sun exposure, food, and supplements are biologically inactive, and are activated through two enzymatic hydroxylation reactions in the liver and kidneys [11].

The vitamin D receptor (VDR) gene is located on chromosome 12 (12q12-14), is composed of 9 exons, and is responsible for mediating the biological actions of vitamin D, it is considered a nuclear receptor that acts as a transcription factor [12–13]. The VDR is widely distributed in human tissues, such as skin and adipocytes, and several factors influence its expression [14]. The main single nucleotide polymorphisms (SNPs) of the VDR gene, i.e., those that cause alteration in only one nucleotide, are ApaI (rs7975232), BmsI (rs1544410), FokI (rs2228570), and TaqI (rs731236) [15]. The TaqI polymorphism is located in exon 9, at the 3’ end of the VDR gene, corresponding to the substitution of the nucleotide thymine (T) for cytosine (C), and its action transforms the adenine-thymine-thymine (ATT) codon into adenine-thymine-cytosine (ATC). This alteration is known as silent alteration, since both ATT and ATC will form the amino acid isoleucine; however, this alteration can affect splicing, mRNA stability, and VDR translation [16–19].

Adipose tissue, in addition to expressing VDR, also acts by storing vitamin D, which, in turn, helps regulate adipogenesis, interfering with the expression of adipokines and reducing inflammation generated in adipose tissue [20]. However, obesity is associated with low-grade inflammation, which can develop into a systemic inflammatory state [21]. Thus, low vitamin D levels can lead to an inflammatory state in adipose tissue [22]. In the literature, there is great variability in the association of the TaqI polymorphism allele (rs731236), which is associated with obesity, according to the population studied [23–24]. Therefore, the objective of this study was to evaluate the effect of the TaqI polymorphism and biochemical, inflammatory and oxidative stress parameters in individuals with obesity.

## Methods

### Ethical aspects

The study has been approved by the Research Ethics Committee of the Federal University of Pampa (UNIPAMPA), under approval number 5.308.525 and was performed in accordance with the ethical standards laid down in the 1964 Declaration of Helsinki and its later amendments. Participants signed the Informed Consent Form and a sociodemographic and health questionnaire with pre-established questions, developed by the authors, was applied.

### Study population

The inclusion criteria were: adult individuals aged between 18 and 59 years who voluntarily agreed to participate in the study. Only individuals who did not meet all the requirements of the data collection protocol and those who were using prior vitamin D supplementation were excluded from the study. Participants were invited to participate in the research, with data collection taking place from October 2023 to June 2024. After completing the questionnaire, anthropometric measurements were taken, including weight, height, waist circumference (WC), and neck circumference (NC), in addition to venous blood collection for subsequent analyses.

We obtained a total sample of 280 individuals, initially, to integrate the study; however, 5 were taking vitamin D supplementation and were removed from the study, resulting in a sample of 275 participants. Since the maximum capacity of the extraction kit used was 250 samples, the final number for genetic analysis was 246 individuals, according to the sample size calculation performed using G*Power 3.1 software, the main variable of interest was considered to be BMI and genotypes of the VDR gene TaqI polymorphism. A statistical power of 95% (β = 0.05) was adopted, with significance level of 5% (α = 0.05) and an effect size was expressed using Cohen’s f (f = 0.25), defined based on the conventions proposed by Cohen [25], which corresponds to a medium effect size [25].

Participants were divided into 3 groups according to their Body Mass Index (BMI): normal weight or eutrophic (18.5–24.9 kg/m²), overweight (25–29.9 kg/m²), and obesity (≥ 30 kg/m²), the latter group encompassing obesity grades I (30 to 34.9 kg/m²), II (35 to 39.9 kg/m²), and III (≥ 40 kg/m²). The eutrophic group consisted of 84 individuals, the overweight group had 79 individuals, and the obesity group consisted of 83 individuals. A post hoc power analysis was conducted using G*Power to assess whether the study had sufficient sensitivity for subgroup comparisons, beyond the overall population analysis. The estimated power was 0.88 for the comparison between eutrophic and overweight groups, 0.89 for eutrophic versus obese, and 0.88 for overweight versus obese (α = 0.05, d = 0.50), suggesting adequate sensitivity to detect moderate effect sizes.

### Obtaining and preparing samples

This research was conducted in the Central Health Center laboratory, in the Nursing Room of the Road-Linked Dry Port (land terminal connected by roads or rail to a seaport, where import and export truckloads are stored and cleared by the Federal Revenue Service), and in UNIPAMPA’s Clinical Hematology and Cytology Laboratory, both located in the city of Uruguaiana, Rio Grande do Sul, Brazil. Ten mL of venous blood were collected from the participants by qualified professionals and placed in tubes with ethylenediaminetetraacetic acid (EDTA) to obtain plasma and with clot activator gel to obtain serum. The materials were refrigerated in a thermal box for transport under appropriate conditions to UNIPAMPA’s Clinical Hematology and Cytology Laboratory, where the samples were processed and centrifuged, separated into aliquots, and stored in a freezer at -20 °C for subsequent analyses.

### Analytical Determinations

#### DNA extraction and amplification of the Taq1 polymorphism of the VDR gene

DNA was extracted from participants, whole blood using Invitrogen^®^ Purelink Genomic DNA kit (Thermo Fisher Scientific^®^, Carlsbad, CA, USA). The VDR gene TaqI polymorphism (rs731236) was genotyped using a StepOne™ RT-PCR (Real-Time Polymerase Chain Reaction) thermocycler (Thermo Fisher Scientific^®^, Waltham, MA, USA), determined by the TaqMan SNP genotyping assay (C__2404008_10 - Applied Biosystems^®^, CA, USA).

#### Biochemical parameters

For the measurement of vitamin D (25-hydroxyvitamin D [25(OH)D]), the electrochemiluminescence method was used, measured using COBAS^®^ e-411 instrument. Vitamin C was measured according to the Jacques-Silva (2001) [26] method, based on precipitation with trichloroacetic acid (TCA), derivatization with 2,4-dinitrophenylhydrazine (DNPH), and color development in acidic medium for spectrophotometric reading. Total proteins were measured using the Labtest^®^ kit (Lagoa Santa, MG, BR), both read on the Biospectro^®^ SP-22 spectrophotometer (Curitiba, PR, BR). All biochemical analyses were performed using serum samples.

#### Inflammatory parameters

For the determination of C-reactive protein (CRP) and interleukins IL-1β, IL-6, IL-8, IL-10 and tumor necrosis factor alpha (TNF-α), an Enzyme-Linked Immunosorbent Assay (ELISA) was performed using serum and kits from Thermo Fisher Scientific^®^ (Waltham, MA, USA). For the measurement of ferritin, the electrochemiluminescence method was used, measured using COBAS^®^ e-411 instrument.

#### Oxidative stress parameters

In the evaluation of lipid peroxidation, the Thiobarbituric Acid Reactive Substances (TBARS) test was used, according to the methodology of Okhawa (1979) [27], based on the reaction of malondialdehyde (MDA) with thiobarbituric acid (TBA) in acidic medium and under heating, quantified by spectrophotometric reading on Biospectro^®^ SP-22 spectrophotometer (Curitiba, PR, BR). To assess oxidative damage to proteins, protein carbonylation was used, according to the methodology of [28], in which carbonyl groups formed during protein oxidation are precipitated with TCA, react with DNPH, and then washed with ethanol/ethyl acetate to remove excess DNPH, where the protein precipitate is solubilized and the absorbance is determined by spectrophotometric reading on Spectramax M5^®^ equipment (Molecular Devices^®^, Sunnyvale, CA, USA). Both analyses used plasma as sample. The endogenous antioxidants Glutathione peroxidase (GPx) and superoxide dismutase (SOD) were measured using commercial kits from RANDOX^®^ (Ransel and Ransod, respectively). Catalase was determined according to methodology [29], based on the decomposition of hydrogen peroxide (H₂O₂) by the enzyme catalase and read on Shimadzu Corporation^®^ (Kyoto, JP) spectrophotometer. Whole blood was used as sample for all antioxidant parameters.

### Statistical analysis

For qualitative variables, results were expressed as absolute frequency (n) and relative frequency (%). For quantitative variables, the normality of the distributions was assessed using Shapiro-Wilk test. For parametric data, a one-way Analysis of Variance (ANOVA) with Bonferroni post-hoc test was performed, and for non-parametric data, the Kruskal-Wallis test followed by Dunn’s post-hoc test was performed, with analyses conducted in triplicate. Results were expressed as mean and standard deviation (SD) for parametric data and as median and interquartile range (IQR) for non-parametric data. Extreme values were handled by imputing the mean, which replaces extreme values with the mean value of the variable, preserving the consistency of the dataset. This approach was adopted due to the low proportion of extreme values, which helps to minimize distortions in the distribution and avoids data exclusion, preserving the central tendency without amplifying the variability. Genotypic frequencies were tested for Hardy-Weinberg equilibrium using the chi-square test in order to ensure that the population is in genetic equilibrium. For categorical variables, the chi-square test was used. Finally, a binary logistic regression was performed using the backward Wald elimination method, with 95% confidence interval, including in the model all variables with significance in the univariate analyses, with obesity as the outcome variable. BMI was adopted as primary variable for sample size calculation because it is the most widely used indicator to classify nutritional status and is directly related to the study’s objective. The TaqI polymorphism was also considered in the analysis, given its biological relevance to the investigated outcome. The other anthropometric parameters were considered secondary variables and analyzed in a complementary manner, as they provide supplementary information to the nutritional status assessment, allowing for a more comprehensive analysis of body composition, but without constituting the primary outcome of the study or serving as basis for sample size calculation.

Statistical analyses were performed using GraphPad Prism version 9.5 (San Diego, CA, USA) and Statistical Package for the Social Sciences (SPSS) version 20.0 (Chicago, IL, USA). The significance level considered was *p* < 0.05.

## Results

### Sample profile according to sociodemographic indicators

The study included 275 individuals, 138 men (50.18%) and 137 women (49.82%). Predominance of participants who self-identified as white (167–62.31%) and those with completed secondary education (75–27.37%) was observed. Regarding family income, the income distribution indicated higher frequency of one to three minimum wages (in 2024, one minimum wage corresponded to 1412 reais), with 90 participants and a percentage of 47.87%. The largest proportion of the sample in terms of marital status was single, with 158 participants (57.66%). The practice of physical activity, smoking, and alcohol consumption were assessed by self-report and classified dichotomously (yes or no), with alcohol consumption being predominant (160 participants, 58.18%), followed by non-smoking (237 participants, 88.43%) and non-practice of physical activity (145 participants, 52.73%). The average age of the participants was 38.52 ± 15.06 years, weight 81.45 ± 19.10 kg (kg), height 168.30 ± 9.15 centimeters (cm), BMI 28.73 ± 6.19 kg/m2, WC 97.11 ± 16.83 and CP 38.90 ± 6.25 cm.

In subsequent analyses, the sample size was restricted to 246 individuals due to the maximum capacity of 250 samples of the extraction kit used, ensuring the integrity and uniformity of the processing. To establish a comparison, the sample was stratified into 3 groups according to BMI: eutrophic, overweight, and individuals with obesity. The continuous variables (age, weight, BMI, WC, and PC) were significantly higher in the obesity group when compared to the other groups (*p* < 0.001), as expected according to the BMI-based classification.

### Distribution of VDR TaqI (rs731236) polymorphism genotype

The allelic and genotypic frequencies of VDR gene TaqI polymorphism (rs731236) are presented in Table 1. The studied population is within Hardy-Weinberg equilibrium (χ² = 0.32, *p* = 0.57), then there was no significant difference in the distribution of the TaqI genotype among the BMI groups. The genotype was stratified, the studied groups were separated according to BMI. We can observe that the most frequent genotype in the eutrophic and with obesity groups was TC, while in the overweight group it was TT.

**Table 1:** Allelic and genotypic frequency of VDR gene TaqI polymorphism (rs731236) in the total sample and in the groups

TaqI Polymorphism		Total (*n* = 246)		(%)		∗ *p* value					
*Genotype frequency*											
TT		102		41.46				0.42			
TC		116		47.15				0.45			
CC		28		11.38				0.12			
*Allelic frequency*											
Allele T		320		65.04							
Allele C		172		34.96							
*Model*											
TC + TT		218		88.62							
CC		28		11.38							

TaqI Polymorphism	Eutrophic (*n* = 84)		Overweight (*n* = 79)		Obesity (*n* = 83)					∗ *p* value	
	n – (%)										
*Genotype frequency*											
TT	30 (29.41)		37 (36.27)		35 (34.31)					0.3194	
TC	43 (37.07)		31 (26.72)		42 (36.21)						
CC	11 (39.29)		11 (38.29)		6 (21.42)						
*Allelic frequency*											
Allele T	103 (32.19)		105 (32.81)		112 (35.00)						
Allele C	65 (37.79)		53 (30.81)		54 (31.40)						
*Model*											
TC + TT	73 (33.49)		68 (31.19)		77 (35.32)						
CC	11 (39.29)		11 (38.29)		6 (21.42)						

Table 1 presents the allelic and genotypic frequency of VDR gene TaqI polymorphism in the total sample and in the studied groups. The studied population is within Hardy-Weinberg equilibrium (χ² = 0.32). *p-values < 0.05 were considered statistically significant.(Chi-square test).

### Vitamin D and genotype

**Fig. 1:**
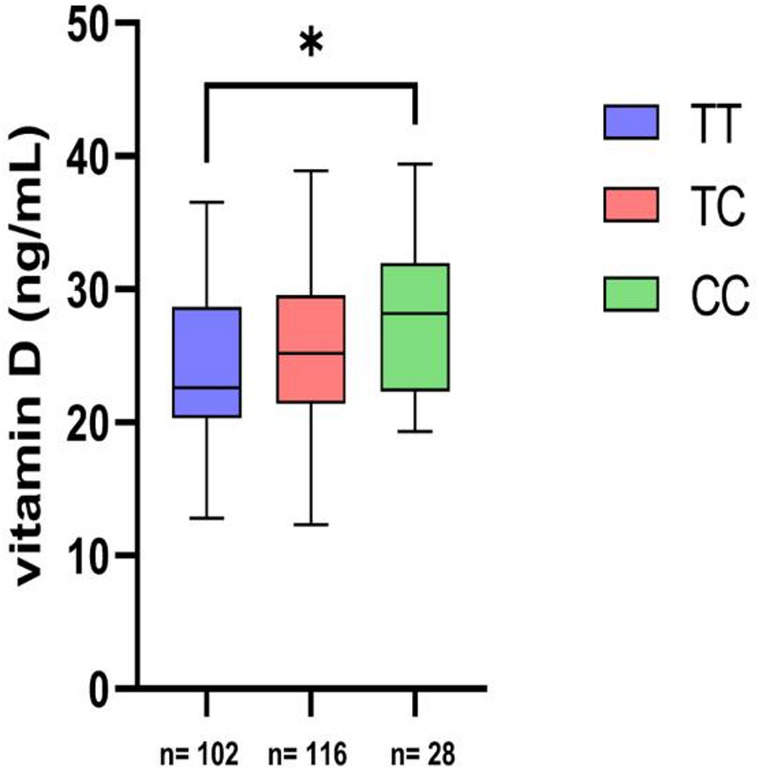
shows vitamin D levels and their relationship to genotype. We can observe that there is increase in vitamin D levels in the CC genotype when compared to the TT genotype (*p* = 0.0172)

Figure 1. Vitamin D levels by genotype. X-axis: genotype groups, also displays the sample size (n) for each genotype; Y-axis: serum vitamin D levels. Data are shown as median (IQR). *p-values < 0.05 were considered statistically significant (Kruskal–Wallis with Dunn’s post hoc test).

### Biochemical parameters

Table 2 shows the biochemical parameters analyzed. Vitamin D levels were higher in the eutrophic group compared to the individuals with obesity group in the TC genotype (*p* = 0.0039). Regarding vitamin C levels, we observed decrease in the levels of this vitamin in the individuals with obesity group when compared to the eutrophic group of TT (*p* = 0.0040) and TC (*p* < 0.0001) genotypes. In the analysis of total proteins, there was increase in protein levels in the eutrophic group when compared to the individuals with obesity group in TT genotype (*p* = 0.0154). In CC genotype, there was increase in protein levels in the eutrophic group compared to the overweight and obesity groups (*p* < 0.0001).

**Table 2:** Biochemical parameters

Parameter	Genotypes TaqI Polymorphism	Eutrophic (*n* = 84)	Overweight (*n* = 79)	Obesity (*n* = 83)	∗ *p* value
*Mediana + IIQ*					
Vitamin D	TT	22.45 (19.40–28.70)	25.80 (21.00–29.40)	22.20 (20.30–28.70)	0.0842
(ng/mL)	TC	26.30 (21.90–31.50)	24.90 (21.40–29.40)	24.60 (20.60–28.50)	0.0039
	CC	28.90 (22.10–35.40)	28.50 (24.60–30.40)	24.75 (20.10–30.20)	0.0892
Vitamin C	TT	8.05 (6.64–10.20)	8.24 (4.93–9.81)	6.93 (5.49–8.61)	0.0040
(mg/L)	TC	9.01 (6.85–10.86)	7.34 (5.91–10.25)	7.43 (5.82–9.35)	< 0.0001
	CC	6.03 (4.51–7.42)	8.52 (4.66–10.56)	6.94 (5.42–7.95)	0.1844
Total	TT	9.87 (8.00–11.68)	9.11 (6.22–11.65)	8.61 (6.66–10.06)	0.0154
proteins	TC	8.59 (6.14–11.18)	10.19 (5.97–12.13)	8.15 (5.89–11.35)	0.7167
(g/dL)	CC	12.15 (10.14–13.08)	9.77 (4.06–11.55)	8.48 (7.58–9.36)	< 0.0001

Table 2 presents biochemical parameters of VDR gene TaqI polymorphism in the studied groups. Data are shown as median (IQR). *p-values < 0.05 were considered statistically significant.(Kruskal Wallis with Dunn’s post hoc test).

### Inflammatory parameters

Table 3 presents the quantified inflammatory parameters. CRP, IL-1β, IL-8, IL-10, and ferritin showed similar pattern, with higher levels in the individuals with obesity group compared to the overweight and eutrophic groups, regardless of genotype, while IL-6 and TNF-α, in some genotypes, showed higher levels in the overweight group compared to the obesity group (*p* < 0,0001).

**Table 3:** Inflammatory parameters

Parameter	Genotypes TaqI Polymorphism	Eutrophic (*n* = 84)	Overweight (*n* = 79)	Obesity (*n* = 83)	∗ *p* value
*Mediana + IIQ*					
CRP	TT	0.80 (0.60–0.90)	1.45 (1.20–2.07)	2.40 (2.10–2.90)	< 0.0001
(mg/dL)	TC	0.80 (0.60–0.90)	1.40 (1.20–2.00)	2.30 (2.10–2.70)	< 0.0001
	CC	0.80 (0.50–1.00)	1.50 (1.10–2.20)	2.45 (2.30–2.50)	< 0.0001
IL-1β	TT	17,50 (14.60–20.30)	30,80 (30.13–33.18)	40.20 (33.50–45.60)	< 0.0001
(pg/mL)	TC	18.90 (15.60–21.30)	30.90 (29.60–33.20)	36.50 (32.60–41.20)	< 0.0001
	CC	15.90 (12.60–18.90)	29.70 (28.80–32.10)	34.05 (33.20–36.20)	< 0.0001
IL-6	TT	9.20 (8.20–11.00)	12.30 (11.20–13.43)	11.30 (10.40–12.30)	< 0.0001
(pg/mL)	TC	9.90 (8.50–11.20)	12.60 (10.90–14.60)	11.70 (10.90–12.40)	< 0.0001
	CC	7.00 (6.60–10.20)	12.10 (10.40–12.30)	12.45 (10.60–12.60)	< 0.0001
IL-8	TT	21.00 (16.00–26.00)	52.00 (42.25–58.75)	63.00 (56.00–74.00)	< 0.0001
(pg/mL)	TC	22.00 (18.00–24.00)	54.00 (41.00–59.00)	65.00 (58.00–69.00)	< 0.0001
	CC	20.00 (16.00–24.00)	55.00 (48.00–65.00)	68.00 (66.00–69.00)	< 0.0001
IL-10	TT	20.50 (14.00–22.00)	39.00 (32.25–41.00)	55.00 (45.00–66.00)	< 0.0001
(pg/mL)	TC	16.00 (14.00–22.00)	37.00 (33.00–41.00)	55.00 (44.00–62.00)	< 0.0001
	CC	21.00 (17.00–22.00)	33.00 (31.00–38.00)	43.50 (41.00–56.00)	< 0.0001
TNF-α	TT	115.5 (104.0–125.0)	156.5 (144.0–187.0)	149.0 (136.0–166.0)	< 0.0001
(pg/mL)	TC	115.0 (99.00–125.0)	158.0 (148.0–166.0)	158.0 (129.0–165.0)	< 0.0001
	CC	114.0 (106.0–136.0)	158.0 (124.0–169.0)	171.5 (158.0–187.0)	< 0.0001
Ferritin	TT	98.50 (84.00–104.0)	325.0 (304.5–357.5)	366.0 (314.0–445.0)	< 0.0001
(ng/mL)	TC	98.00 (85.00–112.0)	332.0 (300.0–420.0)	375.0 (332.0–440.0)	< 0.0001
	CC	93.00 (84.00–101.0)	347.0 (302.0–369.0)	393.0 (358.0–411.0)	< 0.0001

Table 3 presents the inflammatory parameters of VDR gene TaqI polymorphism in the studied groups. Data are shown as median (IQR). *p-values < 0.05 were considered statistically significant (Kruskal Wallis with Dunn’s post hoc test).

### Oxidative stress parameters

Table 4 shows the oxidative stress parameters obtained. TBARS levels were higher in the individuals with obesity group compared to the overweight and eutrophic groups in TT and TC genotypes (*p* < 0.0001), while in CC genotype, levels were higher in the overweight group compared to the obesity and eutrophic groups (*p* = 0.0011). In the protein carbonylation assessment, there is increase in the levels of oxidative damage to proteins in the overweight groups when compared to the eutrophic group in TT (*p* = 0.0230) and TC (*p* = 0.0214) genotypes. In CC genotype, there is increase in the levels of the overweight group compared to the eutrophic and individuals with obesity groups (*p* < 0.0001).

Regarding antioxidant levels, in the GPx analysis, increase is observed in the overweight group in TC genotype compared to the eutrophic group (*p* = 0.0039). In CC genotype, increase is observed in both the overweight and individuals with obesity groups when compared to the eutrophic group (*p* < 0.0001). In the SOD analysis, there is increase in this antioxidant enzyme in the individuals with obesity and overweight groups compared to the eutrophic group in TT (*p* = 0.0022) and CC (*p* < 0.0001) genotypes. In the CAT analysis, we can observe increase in the levels of this enzyme in the individuals with obesity and overweight group when compared to the eutrophic group in TT genotype (*p* = 0.0002). In CC genotype, there is increase in the obesity group when compared to the eutrophic and overweight groups (*p* = 0.0029).

**Table 4:** Oxidative stress parameters

Parameter	Genotypes TaqI Polymorphism	Eutrophic (*n* = 84)	Overweight (*n* = 79)	Obesity (*n* = 83)	∗ *p* value
*Mediana + IIQ*					
TBARS	TT	32.93 (28.55–38.14)	35.95 (26.15–42.62)	40.64 (34.59–51.16)	< 0.0001
(nmol/MDA/ml)	TC	35.32 (19.49–43.03)	36.78 (28.97–49.29)	41.52 (34.39–54.50)	< 0.0001
	CC	35.74 (25.53–44.60)	43.03 (33.55–60.02)	30.11 (22.30–39.28)	0.0011
Carbonyl	TT	1.27 (0.16–2.24)	2.08 (0.27–3.11)	1.63 (0.19–3.76)	0.0230
(nmol/g protein)	TC	2.53 (0.69–3.78)	1.88 (0.12–3.52)	1.43 (0.19–3.66)	0.0214
	CC	0.15 (0.09–0.56)	1.89 (1.09–4.92)	0.27 (0.10–2.28)	< 0.0001
GPx	TT	342.0 (267.5–601.0)	364.6 (264.5–524.0)	343.7 (194.0–525.8)	0.5732
(UI/g protein)	TC	327.5 (215.6–478.7)	461.2 (269.0–807.9)	359.6 (262.9–605.7)	0.0039
	CC	174.6 (145.9–280.0)	379.2 (266.1–1728.0)	411.7 (213.5–717.3)	< 0.0001
SOD	TT	4063 (1755–8560)	7414.0 (2982–15702)	5033 (2843–20195)	0.0022
(UI/g protein)	TC	5409 (2280–17933)	5990 (2875–12890)	6472.0 (4040–13208)	0.3445
	CC	2193 (1386–2980)	14,850 (2778–24405)	10,492 (9387–16014)	< 0.0001
CAT	TT	12,875 (11122–19177)	15,451 (13231–22072)	18,362 (13677–23615)	0.0002
(UI/g protein)	TC	15,568 (12455–23531)	14,563 (8505–26099)	15,777 (11609–22988)	0.8645
	CC	14,566 (9839–21026)	14,249 (11846–22295)	21,989 (20208–24808)	0.0029

Table 4 presents the oxidative stress/antioxidant parameters of VDR gene TaqI polymorphism in the studied groups. Data are shown as median (IQR). *p-values < 0.05 were considered statistically significant (Kruskal Wallis with Dunn’s post hoc test).

### Logistic regression

**Fig. 2:**
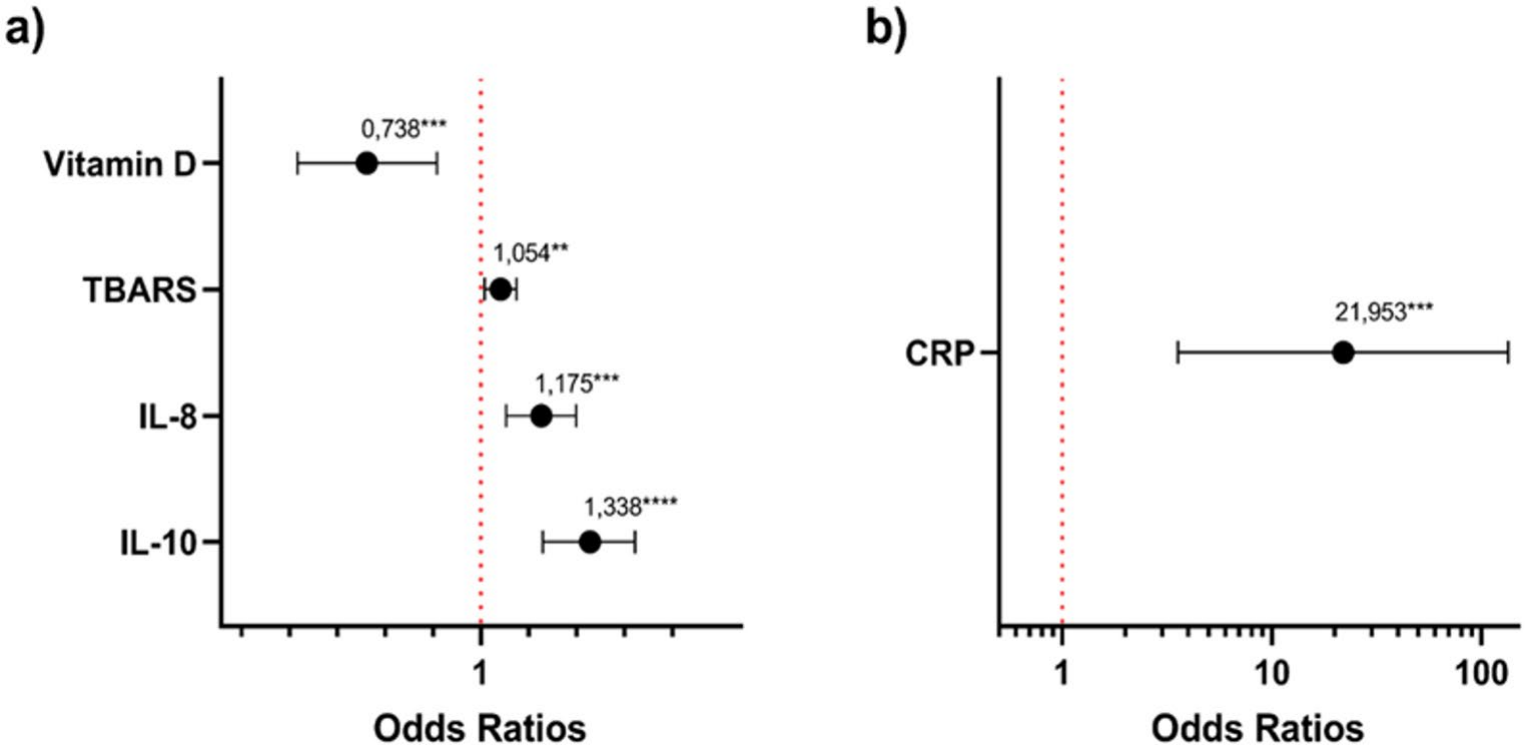
Binary logistic regression (backward Wald) for obesity. The genotype was modeled under a dominant effect (TT + TC vs. CC), with CC as the reference category. The x-axis is presented on a logarithmic scale (log10). In panel (**a**), odds ratios (OR) and 95% confidence intervals (95% CI) are shown for vitamin D, TBARS, IL-8, and IL-10. Panel (**b**) specifically presents the association between CRP levels, displaying the OR and 95% CI for CRP under the same model. The vertical line (OR = 1) indicates no association. Statistically significant associations (*p < 0.05) are indicated

Figure 2 presents the binary logistic regression analysis (backward Wald method). The odds ratio plot was divided into two panels to improve visualization, with vitamin D, TBARS, IL-8, and IL-10 displayed in panel (a), and CRP displayed in panel (b). The variables included in the model were selected considering their potential association with the outcome of obesity. Potential confounding factors (sex and age), the genotype of the polymorphism of interest (TTxTC_CC), and inflammatory and oxidative stress markers (CRP, IL-18, IL-10, and TBARS), as well as vitamin D, were included. The genotype was modeled under a dominant effect (TT + TC vs. CC, reference category). During the selection procedure, sex was removed in the first step, the genotypes TTxTC_CC in the second, and age in the third, not remaining in the final model due to lack of statistical significance according to Wald criterion. The model identified vitamin D (*p* = 0.001) as a significant protective factor for obesity (OR < 1). In contrast, TBARS (*p* = 0.015), IL-8 (*p* = 0.001), IL-10 (*p* < 0.001), and CRP (*p* = 0.001) were significantly associated with increased odds of obesity (OR > 1).

## Discussion

This study demonstrated significant associations among BMI categories and biochemical, inflammatory, and oxidative stress/antioxidant parameters. In addition, binary logistic regression analysis identified vitamin D as independently associated with lower odds of obesity, whereas TBARS, IL-8, IL-10, and CRP were independently associated with higher odds of obesity. Furthermore, we observed a relationship between VDR gene TaqI polymorphism genotypes and circulating vitamin D levels. Together, these findings support the contribution of genetic variability to body weight regulation and its interplay with vitamin D status, metabolic alterations, and inflammatory markers. Our results reinforce the importance of investigating genetic variants in individuals with overweight and obesity to better understand the complex mechanisms underlying metabolic dysregulation.

Regarding the general characteristics of the studied sample, a practically equal distribution between men and women was observed, with predominance of self-declared white participants, with complete secondary education, income of one to three minimum wages (MW) and single marital status. The largest proportion was non-smokers and those that did not practice physical activity, while there was predominance of those who used alcoholic beverages. Considering that MW in 2024 was R$1,412, and that one to three is between 1,412 and 4,236 [30], it corroborates data from IBGE’s National Household Sample Survey (PNAD) of 2024 [31], which shows that in the state of Rio Grande do Sul, the average income was 2,608 reais. In a study carried out in the municipality of Uruguaiana with truck drivers, it was observed that the majority had completed secondary education [32].

Regarding anthropometric data, BMI levels were related to the gradual increase in age, weight, BMI, WC, and PC parameters. A review study demonstrated that aging is related to increase in total adiposity, which may or may not result in increase in body weight [33], and in our study we noted increase in age as BMI increased. The increase in weight, BMI, WC, and PC was expected, as these are parameters that increase according to our stratification of groups in eutrophic, overweight, and individuals with obesity. This weight gain is caused by increased energy intake and decreased energy expenditure, influencing the BMI level [34].

A study by [35] shows that neck circumference (NC) can aid in the assessment of cardiovascular risk, as there is a more pronounced release of fatty acids in the neck region compared to the visceral region. For women, an NC greater than 34 cm signifies an increased risk for cardiovascular disease, and for men, greater than 37 cm [36]. Regarding waist circumference (WC), also considered a marker of predisposition to cardiometabolic events, the reference value is up to 90 cm for men and 80 cm for women [37]. In our study, overweight individuals, women with obesity, and men presented NC and WC above the reference values.

The least frequent genotype for TaqI polymorphism (rs731236) observed in our total population was CC, with 11.38% (Table 1). A study by [38], conducted with a Caucasian (white) Spanish population, demonstrated that the frequency is indeed reduced in the CC genotype, representing only 20% of the population, while in our study it also showed a reduced frequency, with the majority self-declared as white (62.31%). The other genotypes were more balanced in both studies. [23], shows that in their research, carried out in the Greek population, the A allele showed robust association with obesity, and [24], encompassing the population of Saudi Arabia, also related this allele to higher BMI levels. In our findings, the highest frequency of the TC genotype was observed in the eutrophic and individuals with obesity groups, while the TT genotype was more frequent in the overweight group (Table 1).

The study by [39], with black population from South Africa, shows that the TT genotype is associated with higher vitamin D levels compared to the TC and CC genotypes. However, in our study, the CC genotype showed a higher level of vitamin D when compared to the other genotypes; however, it is worth noting that in our study there was prevalence of white population (62.31%). The study by [40], in the Arab population, showed that there was no difference in vitamin D levels in the different genotypes of the TaqI polymorphism. There is a large variability in relation to the populations studied. With our result, we can suggest that the TT genotype affects vitamin D levels (Fig. 1).

The study by Gonzalez-Morelo et al., (2013) [41], conducted in Spain, showed that there is association between low vitamin D levels and greater likelihood of developing some type of obesity, also showing that after weight loss, there was a better response to vitamin D. In our study, vitamin D levels decreased only in the individuals with obesity group compared to the eutrophic group in the TC genotype. Vitamin C levels were found to be decreased in the obesity group in the TT and TC genotypes. Lin et al., [42] [42] showed in their research, conducted in Taiwan, that they assessed whether overweight and people with obesity had deficient intake of micronutrients, including vitamin C, demonstrating that women with obesity had lower adherence to vitamin C intake. In the studies by Madhuvanthi and Lathadevi (2016) [43], in the Indian population, they found no alterations in serum total protein levels associated with BMI, which suggests that adequate levels are being regulated, which does not occur in our study, since the total protein values were increased in the eutrophic group in relation to obesity in the TT and CC genotypes, and also in the latter in comparison with the overweight group (Table 2).

Regarding inflammatory levels, all graphs presented showed increased levels in the individuals with obesity and overweight group compared to the eutrophic group. In some cases, such as IL-6 and TNF-α, the levels in the overweight group were higher than those in the obesity group. The study by Mohamed et al., (2020) [44], conducted with Egyptian women, showed that CRP, IL-6, and TNF-α levels are increased in the obesity groups compared to the normal BMI group, which is consistent with our findings. Furthermore, the study by Ghazizadeh et al., (2024) [45], in the Iranian population, correlated CRP levels with increased probability of being overweight and obesity.

A study conducted in Minas Gerais, Brazil, demonstrated that in both women and men, waist circumference (WC) and body mass index (BMI) were associated with inflammatory cytokines (IL-1β, IL-6, IL-8, IL-10, and TNF-α). However, in men, the cytokines were more strongly correlated, possibly due to the distribution of android fat, as visceral adipose tissue has significant influence on the inflammatory process [46]. In the research by Zaki et al., (2017) [47], an increase in IL-6 and CRP levels was observed in Egyptian women with the polymorphic genotypes TC and CC compared to the wild-type homozygote TT, which we did not observe in our study. Ferritin levels, in both genotypes, show gradual increase with increasing BMI. The study by Shim et al., (2017) [48], in the Korean population, suggested that ferritin is related to abdominal obesity, and is also associated with higher risk of developing metabolic syndrome. A study that evaluated the genotypes of the TaqI polymorphism with ferritin levels, carried out in Egypt, did not show association between these parameters [49] (Table 3).

Thus, these findings highlight the complexity of the interaction between genetic, nutritional, and biochemical factors in overweight and obesity, and reinforce the need for complementary population studies that explore these associations in a comprehensive and stratified manner.

In the study by Caimi et al., (2019) [50], conducted in Italy, TBARS levels and protein carbonylation were elevated in the group with obesity compared to the eutrophic group, where a positive correlation was also observed between these two markers of oxidative stress, demonstrating that the redox state does indeed influence obesity, as found in our study.

Regarding antioxidant enzymes, variances in the results were observed. In their research, Cota Maganã et al., (2024) [51] conducted with Mexican children with obesity and eutrophic weight, GPx and CAT activity was higher in the individuals with obesity group than in the eutrophic group, while in our study GPx was elevated in the overweight group compared to the eutrophic group in the TC and CC genotypes, and CAT, on the other hand, showed increase in the obesity and overweight group in the TT genotype compared to the eutrophic group, and, in the CC genotype, increase in the obesity group compared to the eutrophic and overweight groups, corroborating our findings in this last parameter. In the findings of Brown et al., (2009) [52], in the United Kingdom, they reported that they found no association between SOD levels related to eutrophic, overweight and obesity groups. In our study, in the TT and CC genotypes there was elevation in the obesity and overweight groups compared to the eutrophic group (Table 4).

The results observed in the logistic regression analysis suggest that oxidative stress and inflammation play an important role in the pathophysiology of obesity. On the other hand, vitamin D showed inverse association with obesity (Odds ratio (OR) < 1), and therefore can be identified as an independent protective factor. This finding is consistent with previous studies that relate reduced vitamin D levels to higher body adiposity [41, 53]. Conversely, markers TBARS, IL-8, IL-10, and CRP showed ORs greater than 1, suggesting that elevated levels of these biomarkers are associated with higher risk of obesity. IL-10, a cytokine with anti-inflammatory profile, suggests the hypothesis of a compensatory balance mechanism in response to increased pro-inflammatory cytokines, as shown by Lumeng et al., (2007) [54]. In this context, its levels may reflect this compensatory mechanism in response to the chronic low-grade inflammatory state associated with increased BMI, which may explain its association as a risk factor for obesity in the present study. These results reinforce the role of oxidative stress and inflammation in the pathophysiology of obesity, contributing to the understanding of the complex network of biological factors involved in obesity and indicating potential targets for prevention and intervention strategies. However, longitudinal studies with complementary experimental designs are needed to establish the relationships involved.

### Limitation of the study

One limitation of the study relates to the difference in sample size between the clinical (*n* = 275) and genetic (*n* = 246) analyses. This discrepancy occurred because genetic analysis depended on DNA extraction and successful genotyping, which were limited by the capacity of the extraction kit (maximum of 250 samples per batch) as well as sample quality and processing constraints. Consequently, not all participants with available clinical data could be included in the genetic analyses.

## Conclusion

In this study, higher vitamin D levels were independently associated with lower odds of obesity, whereas increased levels of TBARS (marker of lipid peroxidation), IL-8, IL-10, and CRP were independently associated with higher odds of elevated BMI. Although no direct association was observed between VDR gene TaqI polymorphism (rs731236) and obesity, the TT genotype was associated with circulating vitamin D levels, suggesting that genetic variants may influence vitamin D metabolism or bioavailability, even without a direct relationship with body weight. Overall, these findings highlight the interplay of vitamin D status, inflammatory and oxidative stress markers, and genetic background in the context of obesity. The assessment of inflammatory and antioxidant biomarkers may contribute to improved metabolic risk characterization in individuals with overweight and obesity, while vitamin D emerges as a potential target for preventive and monitoring strategies.

## Data Availability

The authors declare that the data supporting the conclusions of this study are available in the article. Should raw data files in another format be required, these can be obtained upon reasonable request to the corresponding author.
